# Endometriosis and Nutrition: Therapeutic Perspectives

**DOI:** 10.3390/jcm14113987

**Published:** 2025-06-05

**Authors:** Francesco Giuseppe Martire, Eugenia Costantini, Claudia d’Abate, Giovanni Capria, Emilio Piccione, Angela Andreoli

**Affiliations:** 1 Department of Molecular and Developmental Medicine, Obstetrics and Gynecological Clinic, University of Siena, 53100 Siena, Italy; francescogmartire@libero.it (F.G.M.); eugenia.costantini22@gmail.com (E.C.); claudiadabate94@gmail.com (C.d.); 2 SSD Centre for Artificial Nutrition, Clinical and Home Care, Antonio Cardarelli Regional Hospital, 86100 Campobasso, Italy; giovannicapria@yahoo.com; 3 Department of Surgical Sciences, Gynecology and Obstetrics, University of Rome “Tor Vergata”, 00133 Rome, Italy; 4 Program of Gynecology and Obstetrics at Catholic, University “Our Lady of Good Counsel”, 1000 Tirane, Albania; 5 Clinical Program of “Nutritional Sciences” at the Catholic, University “Our Lady of Good Counsel”, 1000 Tirane, Albania; angela.andreoli@uniroma2.it

**Keywords:** diet, endometriosis, inflammation, nutritional factor, painful symptoms

## Abstract

Endometriosis is a chronic, hormone-dependent disorder characterized by an inflammatory response. The disease affects approximately 10% of the general female population, with prevalence rates reaching 30–40% in women with dysmenorrhea and 50–60% in those experiencing infertility. In addition to pelvic pain and reproductive issues, gastrointestinal symptoms, such as acute abdominal pain, constipation, diarrhea, or alternating bowel habits, are frequently reported and can be highly disabling. Emerging evidence indicates that dietary patterns may modulate the inflammatory environment associated with endometriosis, potentially influencing symptom severity by affecting oxidative stress, estrogen metabolism, and levels of sex hormone-binding globulin (SHBG). Diets rich in antioxidants, polyunsaturated fatty acids (PUFAs), and vitamins D, C, and E—alongside the avoidance of processed foods, red meat, and animal fats—may offer beneficial effects. This narrative review explores the relationship between nutrition and endometriosis, emphasizing the therapeutic potential of dietary interventions as a complementary strategy. Notably, dietary approaches may serve not only to alleviate pain and improve fertility outcomes but also to reduce lesion growth and recurrence, particularly in patients seeking pregnancy or those unable to undergo hormonal therapy due to contraindications. Furthermore, nutritional strategies may enhance postoperative recovery and act as a viable first-line therapy when conventional treatments are not applicable. A total of 250 studies were initially identified through PubMed and Scopus. After removing duplicates and non-relevant articles, 174 were included in this review. Our findings underscore the urgent need for further studies to develop evidence-based, personalized nutritional interventions for managing endometriosis-related symptoms.

## 1. Introduction

Endometriosis is a chronic hormone-dependent disease with an inflammatory basis. The prevalence of the disease is around 10% of the general female population, rising to 30–40% of women with dysmenorrhea and up to 50–60% of women with infertility [1]. Symptoms such as pelvic pain and infertility significantly impact social life and the overall quality of life for these women of reproductive age. Less frequent, but equally debilitating, are gastrointestinal symptoms like acute abdominal pain, constipation, diarrhea, or alternating bowel habits that patients with endometriosis report [2].

Deep Infiltrating Endometriosis (DIE) involves endometrial implants infiltrating subperitoneal tissues, typically located in areas like the rectosigmoid, uterosacral ligaments, and bladder. The bowel is the second most affected site, and intestinal endometriosis can cause irritative and obstructive symptoms. While surgery is effective for severe cases, medical treatment is recommended for those without obstructive symptoms to avoid complications such as rectovaginal fistulas and bowel dysfunction [2,3,4,5,6].

Endometriosis is often associated with adenomyosis, which can exacerbate symptoms and further impair fertility [7,8,9,10,11]. Some studies in the literature have suggested that specific dietary patterns may either exacerbate or alleviate the inflammatory state, thereby influencing the severity of painful symptoms. This effect is thought to occur through changes in the levels of free radicals, estrogen, and sex hormone-binding globulin (SHBG) [12].

Multiple theories have been proposed to explain the origins and development of endometriosis, with the metastatic model being the most supported. This theory suggests that endometrial cells, through retrograde menstruation, reach the pelvis and implant [1,13,14,15]. Inflammation, especially caused by ovulation and menstruation, is central to disease progression. Local inflammation triggered by luteinizing hormone (LH) can cause follicular rupture and may contribute to pelvic lesions [2,3,4,16]. Inflammation promotes cellular proliferation and angiogenesis, facilitating the growth of endometriotic lesions [17,18]. Additionally, the immune system’s dysfunction, with elevated macrophage activity and inflammatory mediators like TNF-α, plays a crucial role in lesion survival and progression [19,20]. The persistent inflammatory environment may drive the formation of cystic and deep lesions, further complicating the disease [21,22,23].

Recent research has also indicated that inflammation may influence the body’s response to nutritional interventions. Since chronic inflammation and excessive oxidative stress associated with ectopic lesions play a significant role in the development of endometriosis, nutrients that affect these processes could be pivotal in modifying the course of the disease [1].

The Mediterranean diet, primarily consisting of abundant vegetables, fruits, legumes, seeds, nuts, and moderate amounts of dairy products and fish, with limited red meat and wine [24], has shown promise in providing nutritional support for patients with endometriosis [25]. By reducing the production of pro-inflammatory factors, this diet may help alleviate painful symptoms and potentially reduce disease extension, particularly in cases of DIE in the posterior compartment [26].

This narrative review aims to explore the current understanding of the relationship between endometriosis and nutritional factors, with a particular focus on the potential role of nutrition as a complementary therapy and its impact on symptoms and disease progression. The novelty of the review lies in addressing the role of diet not merely as a supportive or adjunctive therapy but as a potential alternative approach in specific patient populations, particularly in adolescent patients with early-stage or minimal endometriotic lesions. In such cases, where hormonal therapy may not be advisable due to its potential long-term side effects, especially on fertility and development, an early dietary intervention could represent a valuable first-line therapeutic option.

## 2. Materials and Methods

**Figure 1:**
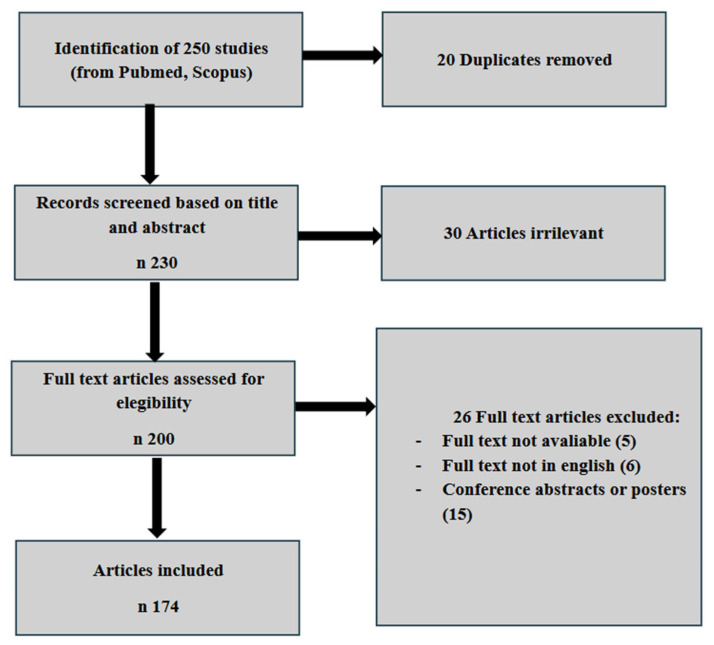
Inclusion criteria diagram.

We conducted an electronic literature search using the MEDLINE database to identify all English-language articles on endometriosis and nutritional factors from inception to June 2024 (Figure 1). Combinations of the following keywords and Medical Subject Headings (MeSH) terms were used to screen and identify relevant studies: “Aliment”, “Adenomyosis”, “Diet”, “Endometriosis”, “Food”, “Feeding”, “Nutrition”, and “Nutritional Factors”, “antioxidants”, “inflammation”, “polyphenols”, “omega-3”, “vitamin D”, “gut microbiota”, “gastrointestinal symptoms”, “vitamins”, “adolescent”. This review included original research articles, such as randomized and non-randomized clinical trials, prospective observational studies, retrospective cohort studies, case–control studies, as well as review articles. Articles were considered eligible if they directly addressed the topic of this narrative review—namely, providing an overview of endometriosis and nutritional factors.

This study was conducted independently by two researchers (F.G.M. and A.A.), who meticulously reviewed all articles meeting the inclusion criteria. All clinical aspects of the topic were discussed, beginning with the pathogenetic theories, followed by an examination of the disease’s prevalence, imaging diagnostics, and concluding with the potential role of nutritional factors as a treatment option.

A total of 250 studies were initially identified through PubMed and Scopus. After excluding 20 duplicate records, 230 articles were screened based on their titles and abstracts, resulting in the removal of 30 irrelevant studies. Of the 200 articles deemed initially eligible for inclusion, 26 were excluded for the following reasons: full text was unavailable for 5 studies, 6 articles were not published in English, and 15 were conference abstracts or posters. As a result, 174 articles were ultimately included in the preparation of this review.

Table 1 lists studies investigating the relationship between endometriosis and nutrition, including a column indicating the strength of each recommendation (Table 1).

The study selection process was conducted in accordance with the PRISMA guidelines. A comprehensive overview of the inclusion and exclusion criteria is provided in the PRISMA flow diagram (Figure 1). Although primarily designed for systematic reviews, the use of PRISMA principles allowed us to transparently define eligibility criteria, handle duplicate records, and document the number and types of studies included, thereby enhancing the methodological rigor and reproducibility of this review.

To ensure transparency and consistency in study appraisal, the quality of the included studies was assessed using the PRISMA checklist, while the Cochrane Risk of Bias (RoB) tool was employed to evaluate potential biases in individual studies.

## 3. Epidemiology and Risk Factors

Accurately estimating the prevalence of endometriosis is challenging due to underdiagnosis. It affects 3–5% of women of reproductive age, increasing to ~30% in infertile women and ~50% in those with pelvic pain [53,54,55]. Subtle endometriosis is found in over 80% of women with pain or infertility, typical lesions in ~50%, ovarian cysts in 25%, and DIE in 1–5%. Superficial peritoneal endometriosis (SPE) is seen in ~40% of asymptomatic patients and in 40–70% of adolescents [2,56,57,58].

Several risk factors have been associated with endometriosis:

- Socioeconomic status

Higher prevalence has been observed among women with elevated socioeconomic status, possibly due to both diagnostic bias and lifestyle-related exposures such as diet, BMI, and physical activity [59,60,61].

- Family history

First-degree relatives of affected women have a 3–9-times higher risk of developing the disease. Twin studies support a genetic contribution similar to other heritable disorders [62].

- Gynecological factors

Early menarche, long/heavy menstrual cycles, and nulliparity are associated with higher risk, likely due to prolonged menstruation. Conversely, multiparity appears protective [63,64].

- Contraception

Combined oral contraceptives reduce menstrual flow and contain progestins, both of which may protect against disease onset or progression.

- Diet

Various pathophysiological processes associated with endometriosis, such as inflammation, estrogen activity, menstrual cyclicity, and prostaglandin metabolism, may be influenced by the diet [65]. These factors, along with some epidemiological studies [66], suggest a potential link between diet and the risk of endometriosis. Current data indicate that a diet rich in vegetables and fruits may have a protective role, while red meat, dairy products, and foods high in unhealthy fats may act as potential risk factors or exacerbators of the disease.

- Physical activity

Regular exercise may lower estrogen levels, improve insulin sensitivity, and raise SHBG, contributing to a reduced risk of endometriosis through anti-inflammatory mechanisms [67,68,69,70].

- Smoke

Smoking increases the mediators of inflammation and can trigger the inflammatory processes associated with endometriosis [71].

- Comorbidities

Gastrointestinal symptoms (e.g., bloating, nausea, diarrhea) are more common in women with endometriosis. Associations with autoimmune diseases like asthma, psoriasis, and RA have also been reported [72,73,74].

## 4. Pathogenesis of Endometriosis

Over the years, several theories have been proposed to explain the origins and development of endometriosis, with the metastatic model being the most widely supported. This model suggests that endometrial cells reach the pelvis via retrograde flow during menstruation and implant there [1,13,14,15]. Both ovulation and menstruation can trigger acute inflammation, which may become chronic if not resolved properly [75,76]. The inflammatory response caused by LH facilitates the rupture of the pre-ovulatory follicle, and this process is disrupted by nonsteroidal anti-inflammatory drugs (NSAIDs) and COX-2 inhibitors, potentially affecting fertility [2,17,18].

The impact of ovulation-related inflammation on ectopic endometrial implants remains uncertain, but it is thought that estradiol released during ovulation may contribute to disease progression [21,22,23]. Hormone therapy after surgery is effective in reducing endometrioma recurrence, with a study showing a significant reduction in cyst recurrence among women who received ovulation suppression [77]. Sampson’s theory of retrograde menstruation [78] explains how endometrial cells migrate into the abdominal cavity and implant in various sites, resulting in endometriotic lesions that can be superficial or deeply infiltrative. The reasons why only a small percentage of women develop these lesions remain unclear, suggesting that other factors, such as immune response and genetic predisposition, play a role [79,80].

Other theories include coelomic metaplasia, which suggests that peritoneal cells transform into endometrial cells due to hormonal or immunological factors [1], and Leyendecker’s “injury and tissue repair” theory, which posits that uterine hyperperistalsis leads to microtrauma and inflammation, promoting the development of endometriosis or adenomyosis [81,82,83,84,85]. Hypoxia-inducible factor (HIF-1α) may play a role in the establishment of endometriotic lesions [86]. The Endometriotic Disease Theory (EDT) [87,88] emphasizes genetic errors in the development of cystic or deep endometriosis, and the genetic/epigenetic theory suggests that oxidative stress and repeated genetic changes contribute to disease progression [89,90].

The embryonic rest theory explains rare cases of endometriosis in women without a uterus, attributing it to remnants of Müllerian duct cells [91,92]. Immune dysfunction also plays a significant role in the survival and proliferation of endometriotic lesions, with activated macrophages contributing to inflammation and angiogenesis [19]. Treatment with anti-TNF therapies has been shown to reduce lesion size in animal models [20].

Endometriosis is commonly associated with painful symptoms, including chronic pelvic pain, dysmenorrhea, dyspareunia, and dyschezia, which worsen during menstruation [93,94]. Infertility rates are also significantly higher among women with endometriosis, affecting 10–30% of patients [95,96,97,98]. Other symptoms include fatigue, bloating, and sleep disturbances [99,100,101,102,103]. If endometriosis is suspected, extra-pelvic symptoms like shoulder pain, pneumothorax, or sciatica should be considered [104]. Unfortunately, a significant diagnostic delay is common, particularly in adolescent girls, who often experience symptoms such as dysmenorrhea from menarche and gastrointestinal issues [1,5,105,106,107,108].

## 5. Posterior DIE and Bowel Functional Symptoms

DIE is characterized by the subperitoneal infiltration of endometrial implants measuring 5 mm or more, typically located in the rectosigmoid, uterosacral ligaments (USLs), rectovaginal septum (RVS), vagina, or bladder [5]. While these implants are often linked to symptoms, such as subfertility, dysmenorrhea, dyspareunia, dysuria, dyschezia, chronic pelvic pain, hematochezia, and hematuria, it often takes 7 to 10 years from the onset of symptoms to reach a diagnosis [109,110].

After the genital tract, the intestine is the second most affected site by endometriosis. Studies suggest that one in ten women with endometriosis has deep bowel lesions [111,112]. Intestinal endometriosis can lead to functional irritative symptoms (e.g., diarrhea, intestinal cramps, hematochezia) due to the release of inflammatory mediators, as well as mechanical obstructive symptoms (e.g., constipation, abdominal bloating) caused by nodules and fibrosis.

Additionally, certain disease localizations trigger specific symptoms; for instance, dyschezia or tenesmus are often associated with endometriosis nodules in the rectum [113,114]. In cases of severe sub-occlusive symptoms, surgery is often the only effective way to relieve the condition. Conversely, for patients without obstructive symptoms, medical treatment presents a viable therapeutic option, especially given the risk of complications from surgery, such as rectovaginal fistulas, anastomosis stenosis, bladder atony, and new-onset bowel dysfunction [6]. The likelihood of surgical complications increases when lesions are extensive, numerous, or located close to the anal sphincter [115,116].

Additionally, patients should be informed that painful symptoms may persist even after surgery [57].

Future studies should investigate whether dietary strategies, such as low-FODMAP, anti-inflammatory, or fiber-modulating diets, may provide symptom relief in women with DIE-associated bowel dysfunction. Given the overlap between gastrointestinal symptoms in DIE and those in functional bowel disorders such as IBS, targeted nutritional interventions could play a crucial role in symptom management. In particular, examining the impact of these diets on visceral hypersensitivity, intestinal inflammation, gut motility, and microbiota composition may yield important insights. Moreover, randomized controlled trials are needed to assess the long-term efficacy, tolerability, and quality-of-life outcomes associated with such nutritional protocols in this specific patient subgroup.

## 6. Discussion: Nutrition as a Therapeutic Strategy for Endometriosis

### 6.1. Nutritional Factor and Inflammation

Recent research has emphasized the strong relationship between inflammation and nutrition. Inflammation is a key factor contributing to disease-related malnutrition, leading to symptoms such as anorexia, reduced food intake, muscle breakdown, and insulin resistance, all of which contribute to a catabolic state. Studies suggest that inflammation can also affect the body’s response to nutritional therapies. Since chronic inflammation and oxidative stress related to ectopic lesions are significant in disease progression, nutrients that influence these processes can modify the course of the disease [1].

In cases of malnutrition, the sympathetic nervous system, immune system, and hypothalamic–pituitary–adrenal (HPA) axis are activated as part of the body’s systemic stress response [117,118]. The activation of the HPA axis leads to the release of stress hormones like cortisol and catecholamines while suppressing hormones regulating sexual and thyroid functions [117]. In malnutrition, the conversion of thyroxine (T4) to triiodothyronine (T3) decreases, a condition known as “low T3 syndrome,” which serves as an adaptive metabolic response to reduce energy expenditure and limit catabolism [119]. Catecholamines and cortisol increase glycogen breakdown and glucose production in the liver while promoting insulin resistance in peripheral tissues and preventing glucose uptake by cells [117]. Pro-inflammatory cytokines, including IL-6, IL-1β, and TNF-α, are released, activating pathways contributing to malnutrition development. These cytokines also affect brain pathways regulating appetite, delay gastric emptying, and promote the breakdown of skeletal muscle [118,120,121,122]. Moreover, interactions between IL-6, IL-1β, and GLP-1 reduce food intake and induce unintended weight loss [123].

Although nutrition has primarily been studied for its role in providing essential nutrients, energy, and metabolic substrates, there has been growing interest in its anti-inflammatory effects [124]. Key nutrients influencing inflammation include the following:

- Polyunsaturated Fatty Acids (PUFAs): Omega-3 fatty acids (e.g., EPA, DHA) have anti-inflammatory properties, while omega-6 fatty acids, found in animal products, are considered pro-inflammatory. Omega-3s have been shown to reduce cardiovascular risk, rheumatoid arthritis, and cancer cachexia [125,126,127,128,129]. Supplementation with omega-3 fatty acids has shown benefits in reducing inflammatory markers and increasing body mass, although evidence remains moderate, with weak recommendations for their use in cancer [130]. Omega-3 supplementation also helps reduce the risk of coronary heart disease [125,126].
- Saturated and Trans Fatty Acids: Trans fatty acids, derived from partially hydrogenated oils, are linked to pro-inflammatory effects and increase oxidative stress [131]. The role of saturated fatty acids in inflammation remains debated, but long-chain saturated fats may promote inflammation, while short-chain fatty acids could have anti-inflammatory effects [124].
- Fiber: Fiber has recognized anti-inflammatory effects [132]. It is fermented by gut microbiota into short-chain fatty acids (SCFAs), which activate immune-regulating pathways, reducing inflammation by inhibiting NF-κB and promoting PPAR-γ [124]. Although direct evidence remains limited, the known anti-inflammatory properties of SCFAs may prove beneficial in attenuating pelvic inflammation and gastrointestinal comorbidities associated with endometriosis [133,134,135].

Fiber also improves the bioavailability of antioxidants and supports gut health [136].

- Added Sugars: High consumption of added sugars amplifies pro-inflammatory effects. Elevated blood glucose levels from sugary foods can form advanced glycation end products (AGEs), which trigger oxidative stress, inflammation, and cell death. The binding of AGEs to the AGE receptor (RAGE) activates NF-κB, modulating gene expression and promoting inflammation. AGEs are implicated in chronic diseases like atherosclerosis and diabetes [137,138].

In conclusion, the relationship between inflammation and nutritional components is dynamic and interdependent. Nutrition influences inflammation, and inflammation affects the body’s response to nutritional interventions. Despite these connections, the optimal use of nutritional therapies for individuals with significant inflammation remains unclear. Future research should focus on personalized strategies to refine the type, dosage, composition, and timing of dietary interventions to enhance clinical outcomes.

**Figure 2:**
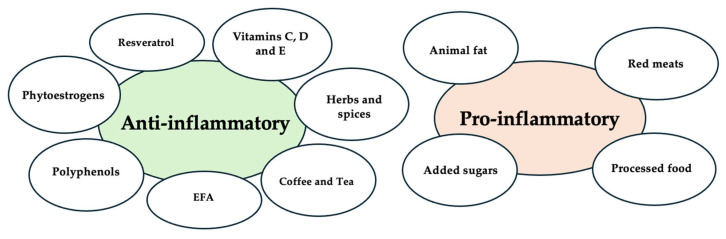
Primary dietary components.

Figure 2 highlights the primary dietary components examined, categorized according to their anti-inflammatory or pro-inflammatory properties.

### 6.2. Nutritional Factor and Functional Bowel Symptoms

The term “functional bowel symptoms” encompasses disruptions in intestinal function, often presenting as irregular bowel habits (constipation, diarrhea, or a mix of both), abdominal bloating, or discomfort. Such symptoms are frequently associated with conditions like irritable bowel syndrome (IBS), a functional disorder where the intestines operate abnormally despite no detectable structural abnormalities. Globally, IBS impacts approximately 4% to 10% of the population at any given time [139,140]. This condition significantly diminishes quality of life, yet current medical treatments often provide limited relief, with outcomes frequently hampered by high placebo response rates [141,142,143]. Even newly developed therapies yield only modest improvements, achieving a 10% to 15% benefit over placebo while incurring substantial costs [144].

Dietary factors play a central role in IBS, with over 80% of individuals reporting that food consumption exacerbates their symptoms [145]. Furthermore, more than 60% of participants in one survey indicated they had modified their diet to manage symptoms [146]. These findings underscore the potential for tailored nutritional strategies to alleviate IBS symptoms and enhance patient well-being.

A prominent dietary intervention for IBS management involves reducing the intake of fermentable oligosaccharides, disaccharides, monosaccharides, and polyols, collectively known as FODMAPs. These compounds are prevalent in various foods, such as specific fruits, vegetables, legumes, and artificial sweeteners. Poorly absorbed FODMAPs like fructose, lactose, and polyols increase water content in the small intestine, while undigested fructans and galacto-oligosaccharides undergo fermentation in the colon, potentially triggering symptoms in sensitive individuals [147,148].

The low-FODMAP diet involves three phases: an initial restriction period lasting four to six weeks, followed by a gradual reintroduction of foods to identify individual tolerance levels, and finally, the personalization of a long-term dietary plan based on those findings [149]. Over the past decade, numerous randomized controlled trials (RCTs) and meta-analyses have demonstrated that the restriction phase effectively alleviates IBS symptoms [150,151,152,153].

In addition to the low-FODMAP diet, national guidelines from organizations like the British Dietetic Association (BDA) and the National Institute for Health and Care Excellence (NICE) recommend practical dietary measures. These include consuming smaller, more frequent meals, maintaining proper hydration, and limiting the intake of caffeine, alcohol, carbonated beverages, and excessive fruit. These approaches are complementary to FODMAP reduction and can support symptom management [154,155].

While the short-term benefits of the low-FODMAP diet are well documented, its long-term impacts remain uncertain. The reintroduction phase, where high-FODMAP foods are carefully reintroduced to assess tolerance, is a critical component of the diet. Research indicates that undergoing this phase with the guidance of a dietitian can lead to sustained symptom relief [156,157]. However, further studies are required to substantiate these results, as logistical challenges often hinder the execution of high-quality trials [158]. Recent recommendations from the British Society of Gastroenterology endorse the low-FODMAP diet as a secondary treatment option for IBS [153].

IBS symptoms frequently overlap with those of endometriosis, a condition that increases the likelihood of an IBS diagnosis two- to threefold in affected women compared to those without endometriosis [26,27,28,29]. This overlap suggests a shared underlying pathophysiology rather than mere coincidence. Both conditions are linked to chronic low-grade inflammation and present with similar clinical manifestations.

In a study involving 160 women with IBS, Moore et al. reported significantly greater symptom improvements among participants with both IBS and endometriosis who followed a low-FODMAP diet compared to those with IBS alone (72% vs. 40%; *p* = 0.001) [26]. These findings highlight the potential of the low-FODMAP diet to alleviate symptoms in individuals managing both conditions [26].

Recent evidence indicates that gut microbiota composition plays a pivotal role in modulating systemic inflammation and estrogen metabolism, both central to endometriosis pathogenesis. Dysbiosis—characterized by reduced microbial diversity and altered SCFA production—has been observed in affected women and may contribute to chronic inflammation and immune dysregulation, favoring lesion growth. Altered gut microbiota may also impact estrogen metabolism via the estrobolome, leading to increased circulating estrogen levels that sustain ectopic endometrial tissue. SCFAs, particularly butyrate, exert anti-inflammatory and immunomodulatory effects, supporting intestinal barrier integrity and reducing systemic inflammatory responses. A compromised gut barrier can increase endotoxin translocation, further promoting pelvic inflammation. Dietary strategies that restore microbial balance and boost SCFA production, such as fiber-rich diets, polyphenol intake, and probiotic or prebiotic supplementation, may, thus, offer symptom relief and limit disease progression. These findings highlight the gut–reproductive axis as a promising target for non-hormonal interventions in the management of endometriosis [159,160].

### 6.3. Nutritional Factor and Endometriosis

Endometriosis is a chronic, estrogen-dependent inflammatory disease that is both complex and multifactorial, with the underlying causes still only partially understood. While current pharmacological therapies can effectively reduce pain and slow disease progression, they do not offer a definitive cure and are not always well tolerated. In this context, personalized and complementary approaches, such as functional nutrition, have gained growing interest in recent years.

Targeted dietary strategies, planned with the support of a qualified nutritionist, may enhance the effects of medical therapies, especially for managing early-stage symptoms, supporting younger patients, or helping individuals who cannot undergo conventional treatments, such as women trying to conceive [30,53].

Conversely, certain dietary habits, such as the frequent consumption of foods rich in pro-inflammatory compounds (e.g., processed meats, red meat, trans fats), have been associated with a higher risk of symptom exacerbation or disease onset in predisposed individuals, likely due to their inflammatory effects [31].

Additionally, the frequent overlap between endometriosis and gastrointestinal symptoms suggests that nutritional adjustments may also help reduce digestive complaints [32,33,161].

The adoption of a targeted nutritional plan not only offers a complementary approach to conventional therapies but also serves as a holistic strategy to reduce inflammation, support hormonal balance, and improve the overall quality of life for individuals with endometriosis.

Key dietary elements potentially contributing to symptom relief include the following:

- Vitamins C and E: These antioxidants may act synergistically to reduce oxidative stress. Vitamin C plays roles in neutralizing free radicals, supporting enzymatic activity, collagen synthesis, and the production of catecholamines and vasopressin [162,163,164,165]. Vitamin E is known for its antioxidant, anti-inflammatory, and anti-angiogenic properties [166,167]. While some studies report no significant association between vitamin E levels and endometriosis [34], others have shown lower serum levels in affected individuals, possibly due to increased antioxidant demand [35]. Reduced vitamin C levels in follicular fluid have also been linked to endometriosis [36]. In animal models, vitamin C supplementation has significantly reduced the size and severity of lesions [37,168]. In humans, combined supplementation with vitamins C and E has been associated with reduced pain, inflammation, and oxidative stress [38,39].
- Polyphenols: They are bioactive compounds abundantly found in fruits, vegetables, and other plant-based foods, known for their potent antioxidant, anti-inflammatory, anticancer, and cardioprotective properties [124,137,169,170]. They exert their anti-inflammatory effects through multiple mechanisms, including the neutralization of reactive oxygen species (ROS), modulation of key inflammatory pathways such as NF-κB and MAPK, and inhibition of cyclooxygenases (COXs) [169]. Additionally, polyphenols contribute to gut health by promoting the growth of beneficial microbial populations, further supporting systemic anti-inflammatory activity [135]. Given these properties, polyphenols may play a valuable role in symptom management and disease modulation in endometriosis [40].
- Phytoestrogens: Found in soy, legumes, seeds, and vegetables, these plant-based compounds mimic estrogen and interact with hormonal pathways. Higher intake has been associated with reduced associations with the presence of endometriosis [41,171,172].
- Resveratrol: A polyphenol found in grapes, berries, and red wine, resveratrol has anti-inflammatory and anti-proliferative properties [172]. Laboratory studies show its ability to suppress inflammatory and growth-related pathways [42,43]. Preliminary clinical research combining resveratrol with hormonal treatments has shown symptom reduction, especially pelvic pain [45], although further studies are needed [45].
- Herbs and spices: Turmeric (rich in curcumin), ginger, and chili peppers contain compounds that regulate inflammation and hormonal activity [46,47,173,174].
- Tea and caffeine: Green and white tea varieties are rich in antioxidants like catechins, which combat oxidative stress [175]. However, excessive caffeine intake may increase the risk of endometriosis, highlighting the importance of moderation [48].
- Essential fatty acids: Omega-3s—found in fatty fish, nuts, and seeds—are well known for their anti-inflammatory properties. Higher omega-3 levels have been associated with reduced endometriosis risk or symptoms [49]. The role of omega-6 is more complex, and maintaining a proper balance may be key [50].
- Vitamin D: Emerging evidence suggests that individuals with endometriosis may exhibit dysregulation in vitamin D metabolism. Several interventional studies have shown that vitamin D supplementation may reduce pelvic pain and improve inflammatory and metabolic parameters in these patients [51,176].
- Red meat and processed foods: High consumption of red meat and processed foods has been associated with increased risk of endometriosis in observational studies. Additionally, such dietary patterns have been linked in the broader literature to elevated levels of inflammatory markers and adverse hormonal profiles, supporting recommendations to limit these foods in favor of plant-based proteins and whole grains [52].

Table 2 summarizes the main food items discussed in this review, detailing their reported effects on endometriosis progression and symptom modulation, either aggravating or alleviating them (Table 2).

### 6.4. Comparison with Previous Studies

Many of the findings mentioned above are consistent with prior studies. The role of antioxidant vitamins C and E in mitigating oxidative stress and inflammation has been widely studied [34,35,36,37,38,39,162,163,164,165,166,167,168], with human and animal models confirming their effect on lesion size and pain reduction [37,38,39,168].

Polyphenols and phytoestrogens have also shown promise in modulating inflammatory and hormonal responses associated with endometriosis [38,39,169,170]. Resveratrol, in particular, has demonstrated anti-proliferative effects in both preclinical and early clinical research [40,41,42,171,172].

The anti-inflammatory roles of curcumin, gingerols, and capsaicin (from chili) have been corroborated in various studies [43,44,45,173], and the antioxidant properties of catechins in green and white tea are well established [46]. However, findings on caffeine remain inconclusive, though some studies caution against excessive intake [174].

Essential fatty acids, particularly omega-3s, are supported by data showing reduced disease risk and symptom burden [47,175], while vitamin D’s role in immune modulation is increasingly recognized [48,49].

Dietary patterns high in red meat and processed products continue to be associated with inflammatory responses and hormonal disturbances in patients with endometriosis [50,160].

### 6.5. Limitations and Strengths of the Study

A key strength of this review is the integrated, holistic perspective that combines clinical evidence with biochemical insights. The proposed nutritional strategies support not only the management of physical symptoms but also aim to improve general health and emotional well-being in women affected by endometriosis.

Nonetheless, many of the data come from animal models or observational studies, limiting the ability to establish causation. Randomized controlled trials (RCTs) examining the long-term impact of specific nutrients or dietary patterns in endometriosis are still scarce.

Individual variability is also a major challenge, as genetic, hormonal, and environmental differences influence how patients respond to dietary interventions. Moreover, the heterogeneity of protocols across studies makes comparisons difficult and hampers the formulation of universal recommendations.

### 6.6. Future Perspectives

While endometriosis cannot be prevented due to its multifactorial etiology and genetic predisposition, it is crucial to understand how lifestyle and nutritional factors may influence its progression. Nutritional strategies may not only help alleviate pain and improve fertility outcomes but also reduce lesion growth and recurrence—especially in patients with reproductive goals or in cases where hormonal treatments are not advisable. Furthermore, diet may support postoperative recovery and serve as a viable first-line intervention when conventional treatments are not suitable. In this context, nutrition may play a strategic role in minimizing long-term pharmacological dependence and improving overall quality of life. To better integrate dietary interventions into clinical management, further randomized controlled trials are needed to clarify the specific effects of various nutrients and dietary patterns. The development of personalized nutritional guidelines—tailored to individual inflammatory markers, hormonal profiles, and gut microbiota composition—could significantly enhance therapeutic outcomes. Ultimately, a multidisciplinary approach involving gynecologists, nutritionists, psychologists, and physical therapists may offer the most effective and sustainable care model for patients with endometriosis.

## 7. Conclusions

Medical and/or surgical management remain central to treatment, but it is important to emphasize the complementary role of dietary interventions. An appropriate diet could significantly alleviate painful symptoms, enhance fertility, and potentially reduce the adverse effects of hormonal therapies, although improvements in terms of imaging findings have not yet been clearly demonstrated. Practical recommendations suggest that patients with endometriosis may benefit from a diet rich in antioxidants, PUFAs, and vitamins D, C, and E, which have demonstrated anti-inflammatory properties. Avoiding processed foods, red meat, and animal fats could further support symptom management and overall disease control. Such dietary modifications could be integrated into personalized therapeutic strategies, empowering patients to actively participate in managing their condition. Key areas for future research include exploring the long-term effects of dietary interventions on clinical outcomes, such as pain reduction, fertility improvements, and disease progression. Additionally, studies are needed to investigate the mechanisms through which specific nutrients modulate inflammation and hormonal balance, as well as the potential interplay between diet, gut microbiota, and endometriosis. Finally, advancing research on personalized nutrition may uncover tailored approaches that maximize the therapeutic benefits of dietary changes for individual patients.

## Figures and Tables

**Table 1:** The studies exploring the relationship between nutrition and endometriosis.

Author/Title	Study Type	Nutritional Therapy	Mechanism	Improvement on Symptoms/Disease	Level of Recommendation
[26] Moore JS, Gibson PR, Perry RE, et al. Endometriosis in patients with irritable bowel syndrome: Specific symptomatic and demographic profile, and response to the low FODMAP diet. Aust N Z J Obstet Gynaecol 2017;57:201–205.	Observational study	Low FODMAP diet	Symptom relief for IBS in endometriosis patients	Yes	B
[27] Wu CY, Chang WP, Chang YH, et al. The risk of irritable bowel syndrome in patients with endometriosis during a 5-year follow-up: a nationwide population-based cohort study. Int J Colorectal Dis 2015;30:907–912.	Population-based cohort study	None	Epidemiological association between IBS and endometriosis	No	B
[28] Chiaffarino F, Cipriani S, Ricci E, et al. Endometriosis and irritable bowel syndrome: a systematic review and meta-analysis. Arch Gynecol Obstet 2021;303:17–25.	Systematic review and meta-analysis	None	Evaluates overlap and comorbidity of endometriosis and IBS	No	A
[29] Heard ME, Melnyk SB, Simmen FA, et al. High-fat diet promotion of endometriosis in an immunocompetent mouse model is associated with altered peripheral and ectopic lesion redox and inflammatory status. Endocrinology 2016;157:2870–2882.	Experimental animal study	High-fat diet	Increased inflammation and oxidative stress	No	C
[30] Della Corte L, Di Filippo C, Gabrielli O, et al. The burden of endometriosis on women’s lifespan: a narrative overview on quality of life and psychosocial wellbeing. Int J Environ Res Public Health 2020;17:4683.	Narrative review	None	Impact on quality of life and psychological wellbeing	No	C
[31] Jurkiewicz-Przondziono J, Lemm M, Kwiatkowska-Pamuła A, et al. Influence of diet on the risk of developing endometriosis. Ginekol Pol 2017;88:96–102.	Review	General dietary influences	Dietary patterns may influence inflammation and hormone levels	Yes	C
[32] Trabert B, Peters U, De Roos AJ, et al. Diet and risk of endometriosis in a population-based case-control study. Br J Nutr 2011; 105: 459–467.	Case-control study	Dietary fat and omega-3	High trans fats increase risk; omega-3 may reduce it	Yes	B
[33] Heilier JF, Donnez J, Nackers F, et al. Environmental and host-associated risk factors in endometriosis and deep endometriotic nodules: a matched case-control study. Environ Res 2007; 103: 121–129.	Case-control study	Diet/environmental exposure	Link between diet/environmental toxins and endometriosis	No	B
[34] Da Broi MG, Jordão-Jr AA, Ferriani RA, Navarro PA. Oocyte Oxidative DNA Damage May Be Involved in Minimal/Mild Endometriosis-Related Infertility. Mol Reprod Dev 2018; 85: 128–136.	Experimental study	None	Oxidative DNA damage in oocytes linked to infertility in endometriosis	No	C
[35] Ekici EI, Guney M, Nazıroğlu M. Protective Effect of Cabergoline on Mitochondrial Oxidative Stress-Induced Apoptosis Is Mediated by Modulations of TRPM2 in Neutrophils of Patients with Endometriosis. J Bioenerg Biomembr 2020; 52: 131–142.	Experimental study	None (pharmacological)	Reduction of oxidative stress in immune cells	Yes	C
[36] Lu X, Wu Z, Wang M, Cheng W. Effects of Vitamin C on the Outcome of in Vitro Fertilization–Embryo Transfer in Endometriosis: A Randomized Controlled Study. J Int Med Res 2018; 46: 4624–4633.	Randomized Controlled Trial	Vitamin C	Improves IVF outcomes via antioxidant activity	Yes	A
[37] Hoorsan H, Simbar M, Tehrani FR, et al. The Effectiveness of Antioxidant Therapy (Vitamin C) in an Experimentally Induced Mouse Model of Ovarian Endometriosis. Womens Health 2022; 18: 174550572210962.	Experimental animal study	Vitamin C	Reduces oxidative stress and lesion size	Yes	C
[38] Amini L, Chekini R, Nateghi MR, et al. The Effect of Combined Vitamin C and Vitamin E Supplementation on Oxidative Stress Markers in Women with Endometriosis: A Randomized, Triple-Blind Placebo-Controlled Clinical Trial. Pain Res Manag 2021; 2021: 5529741.	Randomized Controlled Trial	Vitamin C + E	Reduction of oxidative stress markers and pelvic pain	Yes	A
[39] Santanam N, Kavtaradze N, Murphy A, et al. Antioxidant Supplementation Reduces Endometriosis-Related Pelvic Pain in Humans. Transl Res 2013; 161: 189–195.	Clinical trial	Antioxidant supplementation	Reduction of inflammation and pain	Yes	B
[40] Dull A-M, Moga MA, Dimienescu OG, et al. Therapeutic Approaches of Resveratrol on Endometriosis via Anti-Inflammatory and Anti-Angiogenic Pathways. Molecules 2019; 24: 667.	Review	Resveratrol	Anti-inflammatory and anti-angiogenic pathways	Yes	C
[41] Bartiromo L, Schimberni M, Villanacci R, et al. Endometriosis and Phytoestrogens: Friends or Foes? A Systematic Review. Nutrients 2021; 13: 2532.	Systematic review	Phytoestrogens	Hormonal modulation and symptom relief	Yes	A
[42] Cenksoy PO, Oktem M, Erdem O, et al. A Potential Novel Treatment Strategy: Inhibition of Angiogenesis and Inflammation by Resveratrol for Regression of Endometriosis in an Experimental Rat Model. Gynecol Endocrinol 2014; 31: 219–224.	Experimental animal study	Resveratrol	Inhibits angiogenesis and inflammation in endometriosis model	Yes	C
[43] Yavuz S, Aydin N, Celik O, et al. Resveratrol Successfully Treats Experimental Endometriosis through Modulation of Oxidative Stress and Lipid Peroxidation. J Cancer Res Ther 2014; 10: 324–329.	Experimental animal study	Resveratrol	Reduces oxidative stress and lipid peroxidation	Yes	C
[44] Maia H Jr, DA Silva DM, Haddad C, et al. Advantages of the Association of Resveratrol with Oral Contraceptives for Management of Endometriosis-Related Pain. Int J Women’s Health 2012; 4: 543–549.	Clinical trial	Resveratrol + oral contraceptives	Synergistic reduction of inflammation and pain	Yes	B
[45] Meresman GF, Götte M, Laschke MW. Plants as Source of New Therapies for Endometriosis: A Review of Preclinical and Clinical Studies. Hum Reprod Update 2020; 27: 367–392.	Review	Plant-based compounds	Anti-inflammatory and anti-angiogenic properties	Yes	C
[46] Signorile PG, Viceconte R, Baldi A. Novel Dietary Supplement Association Reduces Symptoms in Endometriosis Patients. J Cell Physiol 2018; 233: 5920–5925.	Clinical study	Multicomponent dietary supplement	Reduction in inflammation and symptom severity	Yes	B
[47] Fadin M, Nicoletti MC, Pellizzato M, et al. Effectiveness of the Integration of Quercetin, Turmeric, and N-Acetylcysteine in Reducing Inflammation and Pain Associated with Endometriosis. In-Vitro and In-Vivo Studies. Minerva Ginecol 2020; 72: 285–291.	In-vitro and in-vivo studies	Quercetin, turmeric, NAC	Anti-inflammatory and antioxidant activity	Yes	C
[48] Kechagias KS, Triantafyllidis KK, Kyriakidou M, et al. The Relation Between Caffeine Consumption and Endometriosis: An Updated Systematic Review and Meta-Analysis. Nutrients 2021; 13: 3457.	Systematic review and meta-analysis	Caffeine	Investigates relationship between caffeine and endometriosis risk	No	A
[49] Hopeman MM, Riley JK, Frolova AI, et al. Serum Polyunsaturated Fatty Acids and Endometriosis. Reprod Sci 2014; 22: 1083–1087.	Observational study	PUFAs	Association between fatty acid profile and endometriosis	No	B
[50] Pereira FEXG, Medeiros FDC, Rocha HAL, Da Silva KS. Effects of Omega-6/3 and Omega-9/6 Nutraceuticals on Pain and Fertility in Peritoneal Endometriosis in Rats. Acta Cir Bras 2019; 34: e201900405.	Experimental animal study	Omega-6/3 and 9/6	Improved pain and fertility parameters in rats	Yes	C
[51] Ghanavatinejad A, Rashidi N, Mirahmadian M, et al. Vitamin D3 Controls TLR4- and TLR2-Mediated Inflammatory Responses of Endometrial Cells. Gynecol Obstet Investig 2021; 86: 139–148.	Cellular study	Vitamin D3	Regulates inflammation via TLR pathways	Yes	C
[52] Yamamoto A, Harris HR, Vitonis AF, Chavarro JE, Missmer SA. A Prospective Cohort Study of Meat and Fish Consumption and Endometriosis Risk. Am J Obstet Gynecol 2018; 219: 178.e1–178.e10. [CrossRef] [PubMed].	Prospective cohort study	Meat and fish intake	Red meat associated with increased risk; fish protective	No	B

**Table 2:** The key dietary elements and their potential effects on endometriosis.

Dietary Element	Mechanism/Properties	Potential Effects on Endometriosis	Future Research Focus
Vitamins C and E	Antioxidant properties; anti-inflammatory and anti-angiogenic effects	Reduce oxidative stress and pain, and improve inflammation markers	Optimal dosages and long-term effects in endometriosis management
Polyphenols	Antioxidant, anti-inflammatory, anti-cancer, and cardiovascular benefits	Alleviate symptoms through reduced inflammation	Identifying specific polyphenol compounds most beneficial for endometriosis
Phytoestrogens	Plant-derived compounds mimicking estrogen; support hormonal balance	Potential protective effect, reducing risk and alleviating inflammation	Long-term impact on disease progression and hormonal balance
Resveratrol	Anti-inflammatory, anti-proliferative properties	Suppresses cell growth and inflammation, potential symptom relief (e.g., pelvic pain)	Confirming clinical efficacy, especially in combination with hormonal treatments
Herbs and Spices	Anti-inflammatory compounds (e.g., curcumin, ginger, chili)	Reducing inflammation and managing symptoms	Optimal dosages and delivery methods for curcumin and other spices
Coffee and Tea	Antioxidants like catechins (especially in green/white tea)	Combat oxidative stress and inflammation	Exploring caffeine’s effects on disease progression and symptom management
Essential Fatty Acids	Anti-inflammatory effects (especially omega-3s)	Reducing inflammation and alleviating symptoms	Investigating optimal omega-3/omega-6 ratio for inflammation reduction
Vitamin D	Supports immune function and inflammation regulation	Reduces pain and other symptoms of endometriosis	Assessing vitamin D supplementation’s effects on endometriosis symptoms
Red Meat and Processed Foods	High in pro-inflammatory components (trans fats, hormones)	Worsens inflammation and hormonal imbalances	Impact of reducing red meat and processed foods on disease progression

## Data Availability

The data that support the findings of this study are available from the corresponding author, E.P., upon reasonable request.
